# Novel Evaluation Metric and Quantified Performance of ChatGPT-4 Patient Management Simulations for Early Clinical Education: Experimental Study

**DOI:** 10.2196/66478

**Published:** 2025-02-27

**Authors:** Riley Scherr, Aidin Spina, Allen Dao, Saman Andalib, Faris F Halaseh, Sarah Blair, Warren Wiechmann, Ronald Rivera

**Affiliations:** 1 School of Medicine, University of California, Irvine School of Medicine, 1001 Health Sciences Road, Irvine, CA, 92617, United States, 1 9498246119; 2 Department of Medicine, Stanford Medicine, Stanford, CA, United States; 3 School of Medicine, Tufts University, Boston, MA, United States; 4 Department of Emergency Medicine, University of California Irvine, Orange, CA, United States

**Keywords:** medical school simulations, AI in medical education, preclinical curriculum, ChatGPT, ChatGPT-4, medical simulation, simulation, multimedia, feedback, medical education, medical student, clinical education, pilot study, patient management

## Abstract

**Background:**

Case studies have shown ChatGPT can run clinical simulations at the medical student level. However, no data have assessed ChatGPT’s reliability in meeting desired simulation criteria such as medical accuracy, simulation formatting, and robust feedback mechanisms.

**Objective:**

This study aims to quantify ChatGPT’s ability to consistently follow formatting instructions and create simulations for preclinical medical student learners according to principles of medical simulation and multimedia educational technology.

**Methods:**

Using ChatGPT-4 and a prevalidated starting prompt, the authors ran 360 separate simulations of an acute asthma exacerbation. A total of 180 simulations were given correct answers and 180 simulations were given incorrect answers. ChatGPT was evaluated for its ability to adhere to basic simulation parameters (stepwise progression, free response, interactivity), advanced simulation parameters (autonomous conclusion, delayed feedback, comprehensive feedback), and medical accuracy (vignette, treatment updates, feedback). Significance was determined with *χ*² analyses using 95% CIs for odds ratios.

**Results:**

In total, 100% (n=360) of simulations met basic simulation parameters and were medically accurate. For advanced parameters, 55% (200/360) of all simulations delayed feedback, while the Correct arm (157/180, 87%) delayed feedback was significantly more than the Incorrect arm (43/180, 24%; *P*<.001). A total of 79% (285/360) of simulations concluded autonomously, and there was no difference between the Correct and Incorrect arms in autonomous conclusion (146/180, 81% and 139/180, 77%; *P*=.36). Overall, 78% (282/360) of simulations gave comprehensive feedback, and there was no difference between the Correct and Incorrect arms in comprehensive feedback (137/180, 76% and 145/180, 81%; *P*=.31). ChatGPT-4 was not significantly more likely to conclude simulations autonomously (*P*=.34) and provide comprehensive feedback (*P*=.27) when feedback was delayed compared to when feedback was not delayed.

**Conclusions:**

These simulations have the potential to be a reliable educational tool for simple simulations and can be evaluated by a novel 9-part metric. Per this metric, ChatGPT simulations performed perfectly on medical accuracy and basic simulation parameters. It performed well on comprehensive feedback and autonomous conclusion. Delayed feedback depended on the accuracy of user inputs. A simulation meeting one advanced parameter was not more likely to meet all advanced parameters. Further work must be done to ensure consistent performance across a broader range of simulation scenarios.

## Introduction

### Background

With the rise of generative artificial intelligence (AI) applications such as OpenAI’s ChatGPT, research into its medical application has expanded. Early investigations assessed whether large language models (LLMs) could pass medical trainee licensing examinations [1-5]. Such studies indicated that LLMs can pass medical exams and possess solid foundations in medical reasoning [3,6-8].

An expanding body of literature has focused on LLMs in medical education, including the perspectives of both students and seasoned clinicians [9-13]. Medical students have shown interest in LLMs and frequently use or intend to use them educationally [14]. Studies have also suggested that ChatGPT can be an effective tool for students when entering the clinical wards [15]. Moving from theoretical, classroom-based instruction to hands-on patient care introduces new challenges [16]. The steep learning curve heightens the need for reliable training tools, and generative AI technologies may satisfy this need.

Current literature suggests a need for rigorous validation of student use of generative AI. Frameworks such as RISE (role, input, steps, expectations; ie, inputting the LLM’s role, anticipated input, required steps, and desired expectations) exist for prompt engineering but have been infrequently applied to medical student LLM use [17]. Initial work established prompts for medical trainees to practice common clinical scenarios in platforms such as ChatGPT [9,18]. These studies established that, given the precise wording of prompts, LLMs can act as an effective simulator of basic clinical scenarios. Despite this promising result, these technologies’ unknown accuracy and precision in displaying desirable parameters limits their broader applicability [19-21].

**Figure 1.**
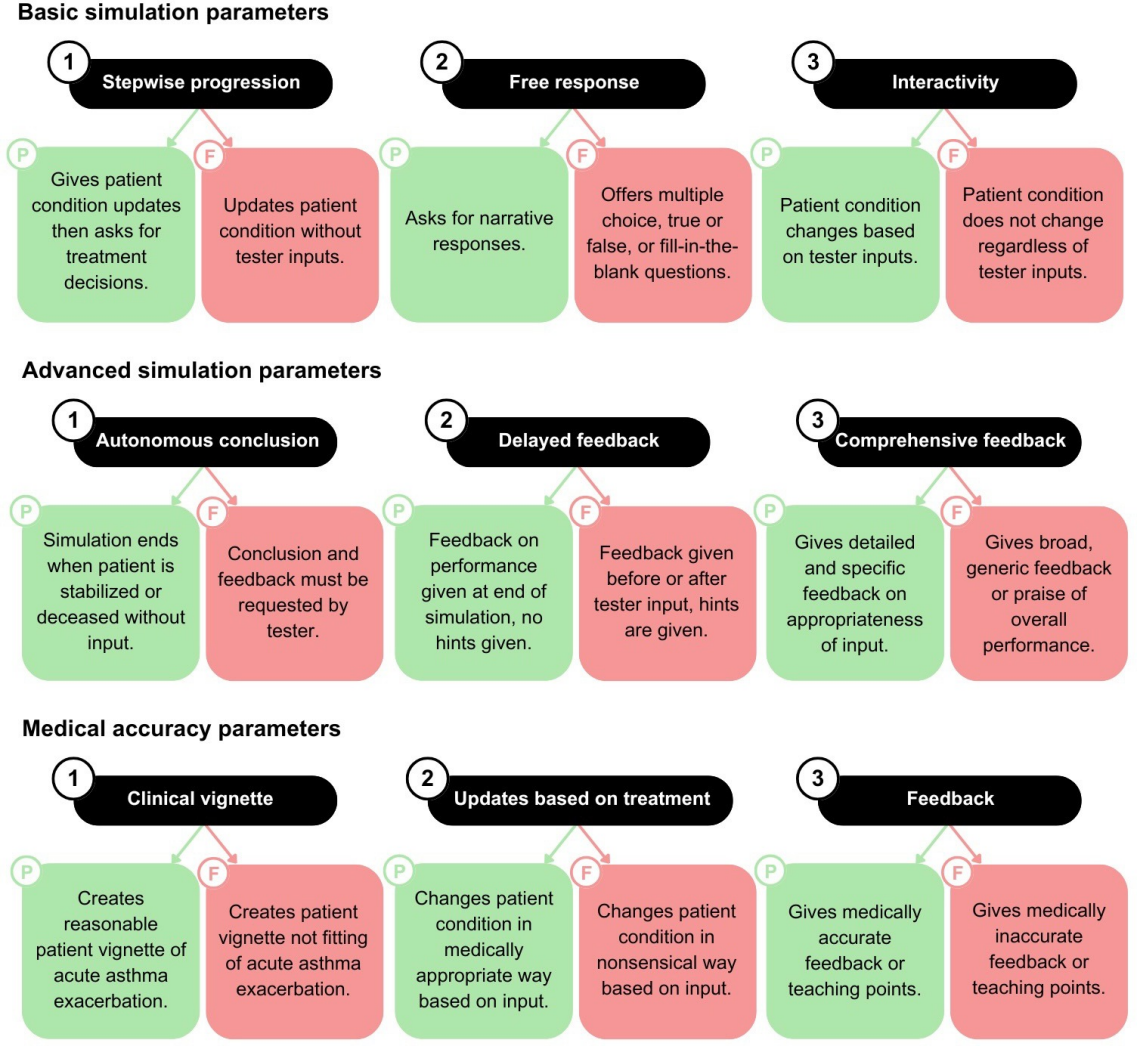
Simulation parameters. Desirable simulation parameters of ChatGPT clinical simulations were based on multimedia educational technology principles and simulation in health care recommendations. Three major parameters were each divided into 3 corresponding subparameters with clear definitions. Subparameters constitute the total 9-part metric by which ChatGPT simulations were evaluated. “P” or green indicates a pass, and “F” or red indicates a fail for whether a simulation exhibits that characteristic.

In this study, we reviewed evidence-based resources for medical simulation and multimedia educational design. Using The Society for Simulation in Healthcare guidelines along with Richard Mayer’s multimedia design principles, we created an evaluation system with 3 main evaluation categories: basic simulation parameters, advanced simulation parameters, and medical accuracy parameters. Each category was further divided into 3 subparameters. Basic parameters were composed of stepwise progression, free response, and interactivity. Advanced parameters were composed of autonomous conclusion, delayed feedback, and comprehensive feedback. Medical accuracy parameters were composed of clinical vignette, updates based on treatment, and feedback. See Figure 1 for definitions.

### Basic Parameters

Stepwise progression in simulation is supported by the segmenting principle of multimedia learning, meaning that learning is maximized when broken into smaller units [22]. At breaks between units, the learner provides the next steps in the simulated patient’s management with reasoning. This is supported by Mayer’s self-explanation principle, which shows strong learning when previous knowledge is integrated into current learning by explaining one’s reasoning [23]. An user-responsive design further facilitates this and creates a generative learning activity poised to improve learning acquisition and retention [24]. These are both in-line with real medical practice where every step of a treatment or diagnosis must be chosen. The free-response format for responses similarly mimics real medical practice, as it allows space for treatment justifications. It is also supported by the active processing principle, which asserts that learning is improved when people actively organize information into cognitive models and integrate prior knowledge to address the new task, rather than passively absorbing teaching points. Just like in real life, where physicians create treatment plans de novo, rather than choosing from a selection of options [25]. Finally, interactivity promotes cognitive engagement and is beneficial to learning outcomes, especially when planned and augmented by peer-to-peer and peer-to-teacher learning [26,27].

### Advanced Parameters

Advanced parameters were selected to improve authenticity and decrease the cognitive load devoted to simulation mechanics, allowing greater focus on learning engagement [28]. An autonomous conclusion is valuable because it provides a specific end point and prevents learners from following extraneous threads when learning goals have been achieved. Learners do not have to decide when they have learned enough, rather they must meet the simulation goals to finish. Delayed feedback keeps novice learners from overrelying on immediate feedback cues to make decisions and promotes active learning in simulations [29]. However, Mayer suggests immediate feedback may be better for some tasks such as solo novice learning or guided reasoning with an expert, but the Society for Simulation in Healthcare guidelines suggest that delayed debrief is most appropriate, and we accordingly chose delayed feedback for our metric [30]. This also mimics the real-life practice of medicine, where the only indication of a treatment’s accuracy is a change in patient stability. Delaying feedback also keeps these simulations in-line with the active-processing principle that active learning is more effective than passive learning from regular performance feedback cues [29]. Finally, comprehensive feedback falls in-line with Mayer’s signaling principle. Signaling, or indicating what information is vital, highlights specific learning points rather than generalizing about a user’s performance [31]. Comprehensive feedback thus ensures meaningful performance assessments with specific takeaways.

### Medical Accuracy Parameters

Medical accuracy is perhaps the most important. This is a common-sense principle that undergirds all the other parameters’ efficacy. Medical accuracy categories were defined whenever there were physiologic, pharmacologic, or pathologic assertions.

A 9-part model for evaluating AI medical simulations is therefore proposed. Using a previously engineered and verified starting prompt, this model helps quantify the consistency of an LLM (ChatGPT-4) in following prompt commands to create simulations exhibiting these 9 features of effective simulations. The provided results aim to better inform recommendations to students and educators who use such resources in their clinical education and establish a method for future evaluation of simulation reliability.

## Methods

### Study Design

The Society for Simulation in Healthcare recommends simulation sessions be high or low fidelity with low physical realism, making AI simulation sessions acceptable when high-fidelity sessions are unavailable [30]. This is also congruent with the authentic learning environments principle which suggests that learning can happen equivalently in any environment as long as the design adheres to effective learning principles [32]. The Society also recommends training should be spaced, frequent, short, and skill-oriented sessions. Therefore, we built our prompt around asthma exacerbation management, a short lesson with evidence-based best practices to be done in 5 to 10 minutes with specific learning takeaways. This is further supported by the Society’s recommendation that each session end with focused feedback via structured debrief, so our prompt accordingly addressed this. The only criteria we were unable to meet were recommendations for in-situ practice and the use of interprofessional teams. However, the authentic learning environments principle again suggests that these simulations can have educational benefits even though they are not in situ, given our use of educational design principles. The following prompt, developed with features of RISE prompt engineering, was subsequently used [9]:

“Please create a clinical scenario on a patient presenting to the hospital with an acute asthma exacerbation and quiz me on what the proper next step of management is. Please make it free response and interactive, meaning you ask me what the next step is one question at a time, and then I write out what I would do, and then you ask me another question based on how my answer would affect the patient. Please update/change the patient’s condition based on my actions, and do not tell me the right answers until the end of the scenario.”

Simulations were run on 1 of 3 ChatGPT-4 accounts due to ChatGPT-4’s token limits and split across Safari, Google Chrome, and Firefox browsers from March 29, 2024, to April 24, 2024. Each simulation was run on its own browser tab session of ChatGPT-4 to eliminate session-dependent memory. All cases simulated an acute asthma exacerbation and focused on treatment steps.

**Figure 2.**
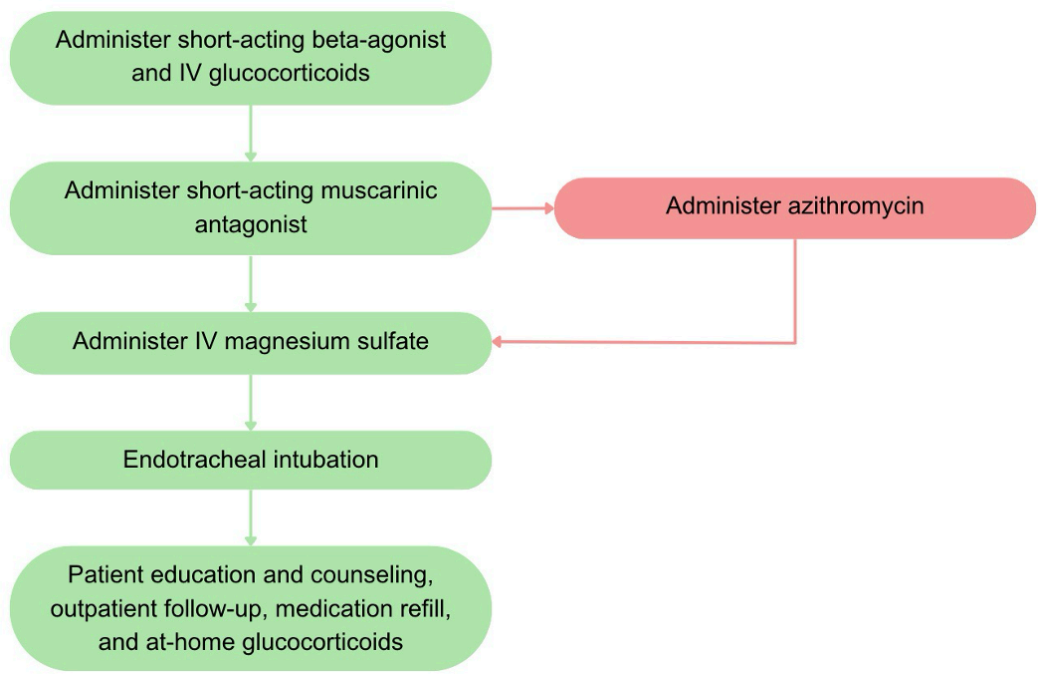
Acute asthma simulation treatment algorithms. Treatment algorithms for acute asthma exacerbation simulations were developed on societal guidelines and were used as a template for ChatGPT simulation inputs. The green pathway represents “correct treatment” and was followed for 180 simulations in the correct subgroup. The red path represents “incorrect treatment,” a variation of the correct treatment algorithm, and was followed for 180 simulations in the incorrect subgroup.

An appropriate treatment algorithm for acute asthma exacerbation was developed based on the review of recommendations from the American Thoracic Society (a leading pulmonology society) and the American Academy of Family Physicians, and confirmed by author RR, a board-certified emergency medicine physician at a high-volume academic medical center [33]. Movement to the next step of the algorithm depended on the simulated patient’s clinical condition, and the tester could proceed to the final step (discharge) if the patient was appropriately stabilized in a stepwise manner. For example, if a patient was no longer in respiratory distress and had normal oxygen saturation after administration of a short-acting muscarinic antagonist, the tester would skip magnesium sulfate and endotracheal intubation and proceed to patient education and counseling. See Figure 2 for treatment workflow.

Simulations were divided into 2 subgroups based on adherence to the treatment workflow. The “Correct Treatment” subgroup followed the treatment workflow. The “Incorrect Treatment” subgroup added administration of a macrolide antibiotic (azithromycin) as the third step (Figure 2). Progression through the algorithm for each simulation depended on the patient’s response to treatment, but all incorrect simulations included steps 1, 2, 3, and 6 at minimum. In total, 180 simulations each were run for the Correct and Incorrect treatment arms, leaving a total of 360 simulations for analysis. Simulations were evaluated according to our 9-part model using the definitions in Figure 1.

Author RS resolved unclear data points. Simulations were reviewed by all authors for medical accuracy and confirmed with author RR, a licensed, board-certified emergency medicine physician with 8 years of clinical experience.

Descriptive statistics were run on GraphPad Prism (version 10.2.3; Graphstats Technologies) and Excel (version 16.85; Microsoft Corp). Statistical significance was determined by *χ*² test, with an α level set at .05.

### Ethical Considerations

Study data were collected by an author (AD) and did not involve personal information or observation of any person’s private or public information. As such data were collected from ChatGPT, all study data were inherently anonymous. This study was reviewed under the University of California, Irvine’s Institutional Review Board Protocol #3213 and deemed not human subjects research and not requiring institutional review board review.

## Results

**Figure 3.**
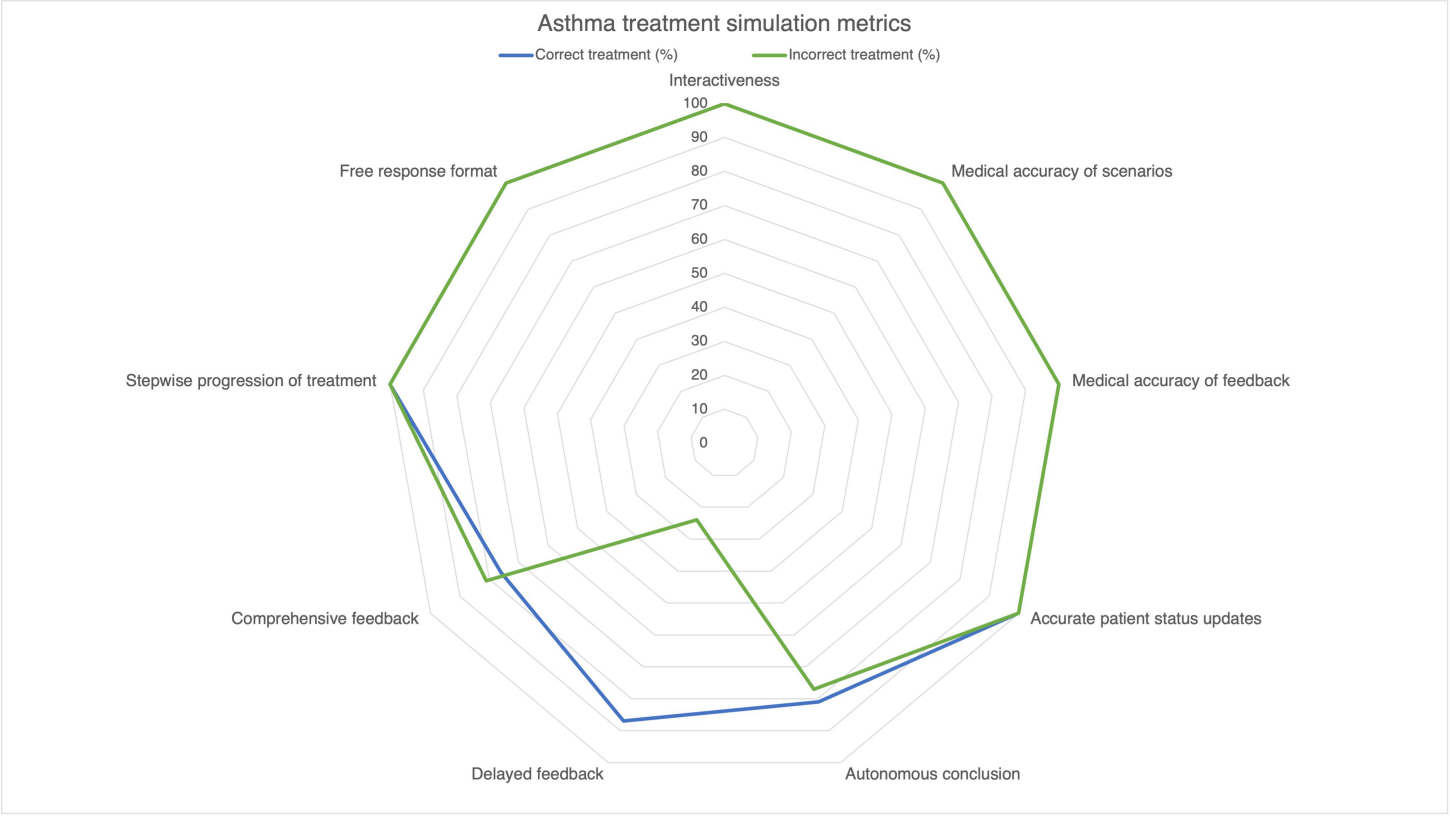
ChatGPT performance on simulation parameters by correct and incorrect inputs. Radar plot showing correct (blue) and incorrect (green) ChatGPT-4 performance on simulation parameters. Each point represents a simulation parameter. Lines indicate the percentage of simulations exhibiting that parameter, with the circumference of the shape indicating 100%. Simulations were given the correct or incorrect acute asthma exacerbation treatment algorithm inputs and evaluated by the 9-part metric developed from multimedia education and simulation principles. Successful exhibition of a parameter was calculated as a percent of all simulations in a subgroup and plotted on the radar plot. Correct and Incorrect subgroups performed similarly on all metrics except “delayed feedback.”

A summary of the results is listed in Table 1 and represented in Figure 3.

**Table 1.** Simulation characteristics^a^.

	Combined (n=360), n (%)	Correct (n=180), n (%)	Incorrect (n=180), n (%)	*χ*² (*df*)	*P* value^b^
Basic parameters	360 (100)	180 (100)	180 (100)	0 (1)	≥.99
Medical accuracy	360 (100)	180 (100)	180 (100)	0 (1)	≥.99
Comprehensive feedback	282 (78)	137 (76)	145 (81)	1.05 (1)	.31
Autonomous conclusion	285 (79)	146 (81)	139 (77)	0.825 (1)	.36
Delayed feedback	200 (55)	157 (87)	43 (24)	146.2 (1)	<.001

a Simulations were given the correct or incorrect treatment algorithm inputs and evaluated on exhibition of characteristics against the 9-part metric. Successful exhibition of a parameter was calculated as a percent of all simulations in a subgroup. The table provides a summary of simulation outcomes.

b *P* values were calculated from *χ*² values

In 360 completed simulations, ChatGPT adhered to basic parameters of stepwise progression of treatment, appropriate patient status adjustments based on treatment inputs, and free response format in 100% (n=360) of simulations. It was also found to be 100% medically accurate in its vignettes, treatment updates, and feedback. There was no difference in medical accuracy between ChatGPT-4 outputs after correct versus incorrect tester treatment responses.

Results for advanced parameters were mixed. Among the correct treatment subgroup (n=180), 87% (157/180) of simulations delayed feedback until the end of the scenario. Only 24% (43/180) of the simulations with incorrect treatment (n=180) delayed feedback (*P*<.001). In all but 2 of these simulations with immediate (nondelayed) feedback, the feedback came after the administration of azithromycin. When combining correct and incorrect simulations (n=360), ChatGPT-4 demonstrated a 55% success rate in delaying feedback.

Simulations with correct treatments concluded the scenario autonomously 81% (146/180) of the time, while those with incorrect treatment concluded autonomously 77% (139/180; *P*=.36). Overall, 79% (285/360) of all simulations, regardless of treatment accuracy, concluded autonomously without tester input.

Correct simulations provided comprehensive feedback 76% (137/180) of the time while incorrect simulations provided comprehensive feedback 81% (145/180) of the time (*P*=.31). Combined analysis showed ChatGPT-4 provided comprehensive feedback on 76% (137/180) of outputs.

Further analysis revealed that ChatGPT-4 was not more likely to conclude the simulation autonomously (*P*=.34) and provide comprehensive feedback (*P*=.27) when feedback was delayed compared to when feedback was not delayed (Table 2).

**Table 2.** Impact of delayed feedback on simulation autonomous conclusion and comprehensive feedback.

	Delayed feedback	Early feedback
Autonomous conclusion^a^	162	123
No autonomous conclusion	38	37
Comprehensive feedback^b^	161	121
No comprehensive feedback	39	39

a Number of all simulations regardless of subgroup that reached an autonomous conclusion versus those that did not based on delayed or early feedback timing.

b Number of all simulations regardless of subgroup that provided comprehensive feedback versus those that did not based on delayed or early feedback timing.

## Discussion

### Basic Parameters

ChatGPT-4 performed perfectly on basic simulation parameters, which is a requirement for it to be a viable educational tool. Its performance agrees with other research on prompt engineering showing proper prompt generation can produce high-quality results [34]. However, even given the use of proper prompt engineering techniques, perfect performance is surprising given ChatGPT’s known inconsistencies [4,34,35]. This may reflect its algorithmic refinement and increased LLM training from widespread use. Given this known feature of LLMs, we should expect it to continue improving over time.

### Medical Accuracy

ChatGPT was also medically accurate for both patient progression within the simulation and in its feedback; simulated patients had reasonable clinical presentations, responded appropriately to therapies, and feedback was in accordance with basic pharmacology, physiology, antibiotic stewardship, and clinical practice. This agrees with other findings on ChatGPT’s clinical reasoning abilities, but the consistently excellent performance is again impressive. However, acute asthma exacerbation is not an exceedingly complex problem and has well-established management guidelines that likely exist in ChatGPT’s training data, so its medical accuracy cannot be generalized to more complex clinical simulations like intensive care management [2,4]. Further research needs to be done into LLM performance differences between complex and simple simulations.

### Advanced Parameters

ChatGPT’s performance on feedback was variable. The observed significant difference in delaying feedback is noteworthy. Delayed feedback best mirrors real-life clinical scenarios where immediate feedback is not always available to redirect incorrect thought processes [36,37]. However, immediate feedback to incorrect answers mirrors the role a teacher might take with a student in simulation, where the teacher does not give a correct answer but instead explains why a learner’s answer was incorrect in an attempt to give a better chance at the correct answer. It is also in-line with the expertise reversal effect that suggests novice learners will do better with more intense educational guidance, while advanced learners do better with less [38]. One possibility is that ChatGPT may be intentionally providing more structured guidance to learners who make errors, recognizing the need for additional support.

Delayed feedback best mirrors real-life clinical scenarios where immediate feedback is not always available to redirect incorrect thought processes. This may be particularly important for learners intent on practicing in smaller community settings where there are fewer opportunities for collaboration. However, this independence is a major developmental process in residency training, and thus, may be above the training level for which these simulations were targeted. Additionally, health care systems are interdisciplinary and collaborative. Expecting completely delayed feedback on patient care may be unrealistic considering shift hand-offs, pharmacists, nurses, medical record alerts, and other checks. Furthermore, the difference between delayed feedback for correct and incorrect treatments may offer different learning experiences for learners of different skills; it is unclear whether these different learning experiences would be inequitable or if they would offer new opportunities for learning (ie, learners who know the proper treatment are ready to practice independently, whereas learners still mastering treatment protocols are not forced to practice independently when they are not ready).

Another consideration is that different prompt engineering could resolve this variability. A prompt could include the instructions to “not give feedback until the end of the scenario, and instead of giving feedback on incorrect answers, make the patient more unstable as a sign that the choice was incorrect, then explain this incorrect choice in the summary feedback at the end of the simulation.” Alternatively, because ChatGPT’s algorithm learns from user inputs, learners running simulations in the same chat window could give ChatGPT feedback as they run simulations to hone in on their desired parameters, including desired feedback mechanisms. Future studies will need to evaluate differential prompt engineering, learner experiences, and learner preferences.

Of note, ChatGPT also gave hints to guide the tester after incorrect inputs. For example, ChatGPT could change from asking about the “next step in management” to asking about the “next step in controlling bronchoconstriction,” thus nudging the tester toward administering a bronchodilator. This feature, as well as not delaying feedback after incorrect inputs, might be ChatGPT exhibiting flow, the educational and gaming principle that tasks should be made difficult enough to optimally challenge the learner, but not so difficult that the learner quits [39]. Again, further studies will need to evaluate learner experiences and preferences.

Comprehensive feedback was not significantly different between correct and incorrect simulations, with an overall rate of 78% (282/360). This high performance is encouraging, as comprehensive feedback offers more detailed learning opportunities and touchpoints for self-study. While this study did not explore the possibility of learners requesting expanded feedback, doing so could potentially increase the rate of comprehensive feedback to close to 100%, thereby enhancing the educational value of the simulations. ChatGPT concluded autonomously at similar rates to comprehensive feedback, which is also encouraging. Autonomous conclusion prevents learners from becoming “trapped” in the simulation, primed by the continuing simulation to believe there is more treatment needed even when the patient is stable and treatment should conclude. While autonomous conclusion at 100% would be best for efficiency and ease of use, forcing a learner to decide when to end the simulation can also mimic the testing style of oral examinations used by some medical specialties for board certification. This is a skill expected of advanced learners who have completed residency, but earlier learners could be easily prompted to end the simulation when they feel they have completed their treatments. Proper instructions and framing could address this problem from the prompt generator’s perspective.

ChatGPT-4 was not significantly more likely to conclude simulations autonomously and provide comprehensive feedback when feedback was delayed. This suggests that ChatGPT-4 does not necessarily perform desirable actions in clusters, meaning that learners who make mistakes are not at risk of losing some of the simulation’s desirable characteristics.

### Future Work

This study focused on a single mistake in the treatment workflow, but future studies should look at performance with multiple mistakes. ChatGPT-4’s ability to display desirable simulation parameters in the presence of multiple mistakes would be crucial for it to be a viable educational tool; real learners may make multiple mistakes of varying severity, and ChatGPT-4 would need to handle this. As is, the feasibility of using this tool given ChatGPT’s varied performance on advanced parameters is still unclear. Medical educators may be hesitant to use a tool that is not completely standardized and risks inequitable learning outcomes, while others may welcome a tool that seemingly adapts to the learner’s level. Quantified student learning gains after simulation use are thus needed for educators to make informed decisions in their classrooms and simulation centers and are the subject of ongoing work. However, with the aforementioned testing, we foresee the following potential implementation: educators would use the most advanced version of ChatGPT available and tailor the prompt provided here to their students’ learning goals. Simulations would focus on simple diagnostic or therapeutic problems with straightforward algorithms. Simulations would be paired with robust pre- or postsimulation didactics to reinforce simulation concepts and not be the sole method of instruction. Ideally, simulations would be overseen by an experienced educator capable of monitoring simulations for accuracy.

Future work should focus on measuring student performance after using simulations to assess educational utility, trialing simulations on different LLMs to assess if the best LLM exists for this purpose, and trialing LLM performance on increasingly complex, nonalgorithmic simulations with multiple wrong answers. All of these are important for this tool’s external validity.

### Limitations

This study has several limitations which should be addressed. First, the simulations were limited to a single clinical scenario—acute asthma exacerbation—which may not be representative of ChatGPT-4’s performance across a wider range of medical conditions. More complex scenarios reflecting the breadth of clinical practice and training level cannot be assumed to function as the simulations provided here. Our simulations were also run on only 1 prompt to limit confounders, but different prompts may produce varied results. Additionally, the study only compared correct treatment and 1 type of incorrect treatment, excluding a broader spectrum of clinical inaccuracies that may occur in real-world educational settings. Furthermore, simulations were run on 3 different ChatGPT-4 accounts and across different browsers, which, although necessary due to token limits, might introduce variability in the model’s responses. Finally, the results of this study are contingent on the current application program interface settings established by ChatGPT and each new update of the LLM can potentially impact reproducibility. However, this does not detract from this study’s conclusions given its intent to establish the methodology for evaluating simulations, rather than being a definitive recommendation for implementing LLM into preclinical medical education.

### Conclusions

The 9-part model described here can be useful in evaluating ChatGPT simulations as a learning tool in accordance with established principles of medical simulation and multimedia design in educational technology. The use of this model can help standardize the evaluation of LLM simulation research. As an example use of this model, ChatGPT performed well in creating practice clinical scenarios that adhered to 3 simulation parameter categories. It performed excellently on 2 primary features, medical accuracy and basic simulation parameters, and reasonably well on advanced parameters. Variation in simulation characteristics based on the accuracy of learner inputs is a point of concern. This tool’s impact on student learning is an important next step to explore, but these simulations demonstrate ChatGPT’s potential to be a reliable educational tool for the appropriate preclinical-to-clinical learning level.

## Supplementary material

10.2196/66478 Multimedia Appendix 1 Supplementary files.

## Abbreviations

AI: artificial intelligence
LLM: large language model
RISE: role, input, steps, expectations
